# Nitro-Perylenediimide: An Emerging Building Block for the Synthesis of Functional Organic Materials

**DOI:** 10.3390/molecules25061402

**Published:** 2020-03-19

**Authors:** Lou Rocard, Antoine Goujon, Piétrick Hudhomme

**Affiliations:** Laboratoire MOLTECH-Anjou, UMR CNRS 6200, UNIV Angers, SFR MATRIX, 2 Bd Lavoisier, Angers CEDEX 49045, France; lou.rocard@univ-angers.fr (L.R.); antoine.goujon@univ-angers.fr (A.G.)

**Keywords:** perylenediimide, nitro group, organic materials

## Abstract

Perylenediimide (PDI) is one of the most important classes of dyes and is intensively explored in the field of functional organic materials. The functionalization of this electron-deficient aromatic core is well-known to tune the outstanding optoelectronic properties of PDI derivatives. In this respect, the functionalization has been mostly addressed in bay-positions to halogenated derivatives through nucleophilic substitutions or metal-catalyzed coupling reactions. Being aware of the synthetic difficulties of obtaining the key intermediate 1-bromoPDI, we will present as an alternative in this review the potential of 1-nitroPDI: a powerful building block to access a large variety of PDI-based materials.

## 1. Introduction

Perylenediimide (PDI) derivatives, discovered by Kardos in 1913 [1], have been extensively studied and exploited in many fields. First, taking advantage of their outstanding high chemical, thermal and photochemical stability [2], they have been initially used as red dyes and pigments [3,4], in paints, lacquers and reprographic processes [5], This application results from the strong visible-light absorbing capabilities of PDI, whose UV-vis spectrum displays three characteristic bands spanning from 430 to 550 nm with high extinction coefficient (up to ≈10^5^ M^−1^ cm^−1^). Those optical properties are the consequence of the strong conjugation within its molecular structure corresponding to an electron-rich polyaromatic core, the so-called perylene, substituted with two electron-withdrawing imide groups at the 3,4 and 9-10-*peri*-positions. Besides, PDI derivatives display very high quantum yields of fluorescence (close to 1), which enables their development as fluorescent dyes [6], near-IR dyes [7], molecular switches [8] and dye lasers [9]. In addition to their excellent photophysical properties, the electron-poor character of PDI is highlighted by a first reduction potential around –1 eV vs. the ferrocenium/ferrocene (Fc^+^/Fc) couple. These characteristics make PDI a strong electron acceptor, with low lying LUMOs and high electron mobility; it is recognized among the best n-type semiconductors available to date and provides strong prospects for investigations in organic solar cells (OSCs) as a good alternative to fullerene [10,11,12,13]. Owing to those particular opto- and electrochemical characteristics, PDIs promote photoinduced electron and/or energy transfer processes and were intensively exploited as building blocks to construct light-harvesting arrays and artificial photosynthetic systems [14,15,16,17,18,19,20,21,22]. Finally, besides the evident applications in electronic devices, the interest for those properties is growing in medicine, such as for photodynamic therapy [23], as PDIs have shown biocompatibility [24] when correctly decorated with water-soluble groups.

**Figure 1:**
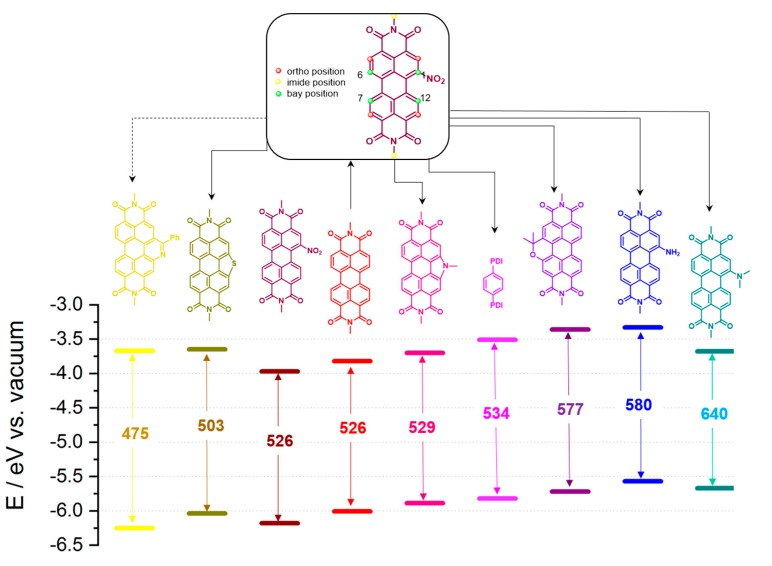
1-NitroPDI, a key intermediate for reaching various bay-substituted-PDI structures with distinct photoredox properties. Frontier orbital energy (HOMO-LUMO) levels of PDIs. Wavelengths (nm) of maximal absorption of light are shown. The data were collected from references [30,31,32,33,34,35].

For all those applications relative to absorption, emission and electron-accepting capabilities, the fine and desired tailoring of the photoredox properties through the tuning of the HOMO-LUMO band gap of PDI is required. Among all the synthetic tools to do so, the most popular methods consist in (1) modifying the size of the conjugated system; (2) changing the planarity of the molecule to affect the π-π interactions by introducing bulky substituents or promoting annulations; (3) inserting electro-donating or withdrawing groups; (4) replacing a carbon atom by a isoelectronic heteroatom (doping method) [25,26,27]. All those strategies have been widely applied to PDI derivatives, mostly through the functionalization of the perylene core at the ortho or bay positions, as modifications at the imide position barely influence the photoredox properties but mostly affect the solubility of the material [28,29]. Historically, the functionalization of the perylene core has been achieved via the formation of key halogenated-PDIs intermediates in the positions 1,12 and 6,7 (bay region); in particular, 1,6,7,12-tetrachlorinated or 1-mono and 1,6/1,7-dibrominated PDIs. In this review, we will present the emergence of nitro-substituted PDIs as new key intermediates for modifying the molecular structure of the PDI core and efficiently tuning its HOMO-LUMO band gap (Figure 1). A particular attention will be given to the monofunctionalization of PDI at the bay position (position 1).

## 2. Nitration vs. Bromination Conditions of Perylenediimide Derivatives

Nitration or halogenation, and more specifically chlorination or bromination, on the perylene core through electrophilic substitution (S_E_Ar) affords key building blocks for further functionalization of PDI derivatives. Such a reaction enables the decoration of the bay positions with respect to Holleman rules, according to the electron withdrawing character of the diimide groups. Historically, the tetra-chlorination of PDIs has enabled the emergence of wide applications by increasing solubility in organic solvents with a significant twisting of the perylene core and the possibility to tune their electronic properties [36]. In this review, we are focusing on the bis, and essentially, the mono-bay-functionalization of PDIs, which limit the twisting of the perylene. To do so, the most popular method is the introduction of bromine atom(s), which can subsequently be replaced through nucleophilic substitution or pallado-catalyzed organometallic coupling reactions. We are herein highlighting that the (mono) nitration of PDI should be considered as an excellent alternative to the (mono)bromination.

**Scheme 1:**
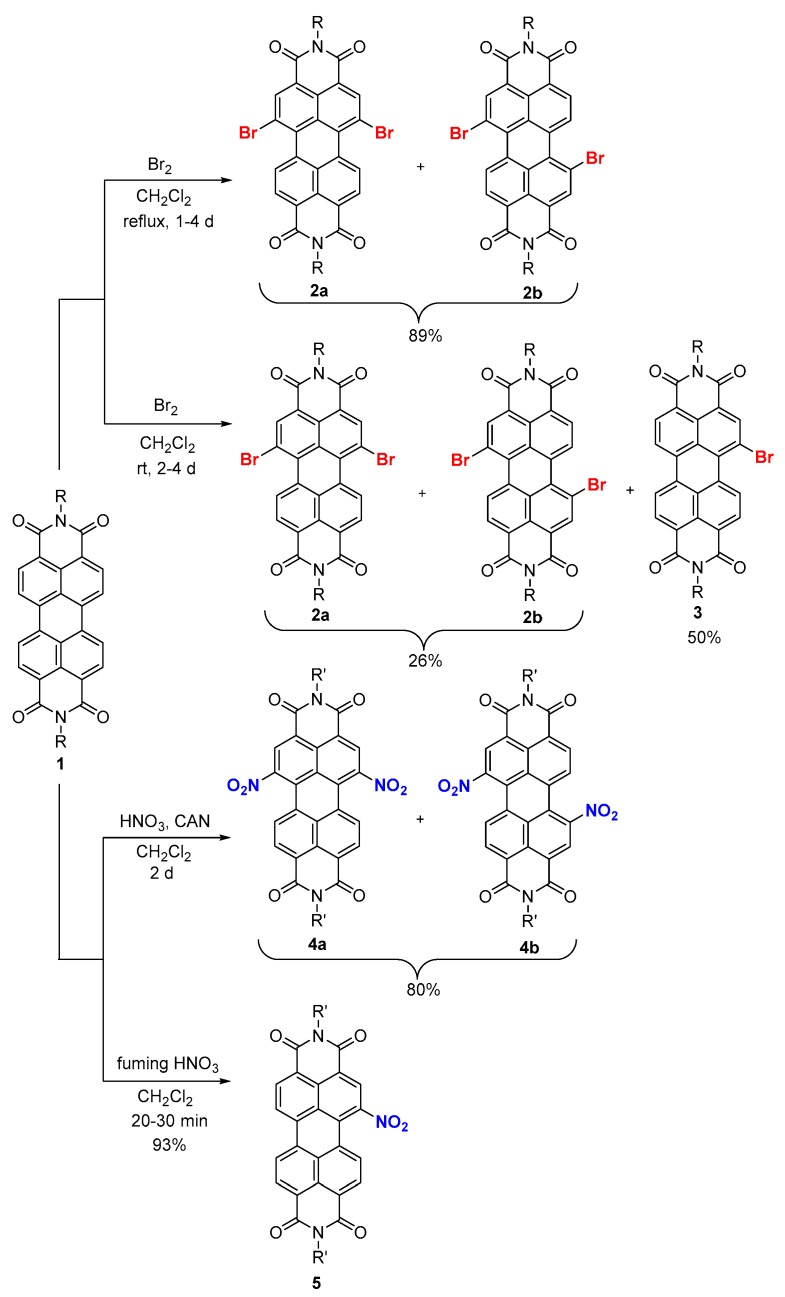
Mono and bis-nitration vs. mono and bis-bromination of PDI. R = 1-ethylpropyl; R′ = cyclohexyl.

For the mono and bis bromination reactions, numerous synthetic strategies have been reported [29]. Nevertheless, the most used pathway using the milder conditions is shown in Scheme 1. Typically, the reactions occur in a chlorinated solvent, in the presence of a large excess (>50 equiv) of bromine at room temperature for more than 2 days to reach 1-bromoPDI **3,** or under reflux for more than 1 day to obtain a mixture of regioisomers 1,6 and 1,7-dibromoPDI **2** in ratio ≈1:3 [37]. The mono-bromination conditions afforded a complicated mixture of dibromo (25%), monobromo (50%) and unreacted PDI requiring fastidious purification by chromatography. Moreover, practically, the quenching of large excess of bromine is a critical step and limits the large-scale use of this essential precursor of PDI-based materials.

The mononitration of PDI **1** is more selective, owing to the electron-withdrawing inductive and mesomeric effects of the nitro group, which sufficiently deactivates the PDI core towards the second electrophilic substitution. The reaction was initially performed by Langhals and colleagues, who attained 98% yield using a solution of N_2_O_4_ gas (prepared by strong heating of Pb(NO_3_)_2_) and methanesulfonic acid (CH_3_SO_3_H) as the catalyst in CH_2_Cl_2_ [38,39], and this was achieved more easily using nitric acid in the presence of cerium (IV) ammonium nitrate (CAN) in CH_2_Cl_2_ with nearly quantitative yields [30,31,40,41,42,43,44,45,46]. The kinetics of the reaction were reported to be higher using a mixture of HNO_3_ and H_2_SO_4_ [47,48,49]. Later, it was shown that the nitration reaction could be carried out using an excess of fuming nitric acid (≈25 equiv), wherein the addition of CAN was not improving the yield or favoring the kinetic of the reaction [32,50]. allowing the preparation of mononitroPDI **5** in 93% yield (at room temperature and in short time) in a multigram scale without the need of purification by silica gel chromatography (Scheme 1) [51]. Under the same conditions, and only by carrying out the reaction for a longer time (6 h), the bis-nitration can occur to afford the two regioisomers (1,6)-and (1,7)-dinitroPDI **4** with no, or almost no selectivity [52]. Notably, when the reaction was described in the presence of CAN for 48 h, the authors claimed a selectivity in favor of the regioisomer (1,7) **4b** (3:1) and the possibility of separating the two regioisomers by HPLC or repetitive crystallizations [40,53]. 

Hence, the functionalization of PDI and in particular its mono-functionalization is far more efficient in terms of yield, time reaction, purification, atom economy, etc., through its nitration than its bromination.

## 3. Reactivity of 1-NitroPerylenediimide

### 3.1. Nucleophilic Substitution of the Nitro Group

The fine and controlled tuning of the electrochemical and optical properties of PDIs is of great interest for engineering fluorophores, color pigments, acceptors in organic transistors and solar cells, etc. To respond to this demand, the synthetic strategy is to attach electron-donating or electron-withdrawing groups on the perylene core. The functionalization with amine and alcohol through nucleophilic substitution is the most common approach. The amino group on the bay position, for instance, drastically changes the optoelectronic properties of PDI, with a long-wavelength charge transfer band, owing to the HOMO located on the amine and the LUMO on the PDI. This strategy has been widely explored using halogenated PDI; in particular, bromo-PDI [29]. As an example, the replacement of bromine atoms in a mixture of 1,6 and 1,7-dibromoPDIs **2** by pyrrolidine moieties was carried out in 80% yield heating the reaction in neat pyrrolidine at 50 °C for 18 h [54].

**Scheme 2:**
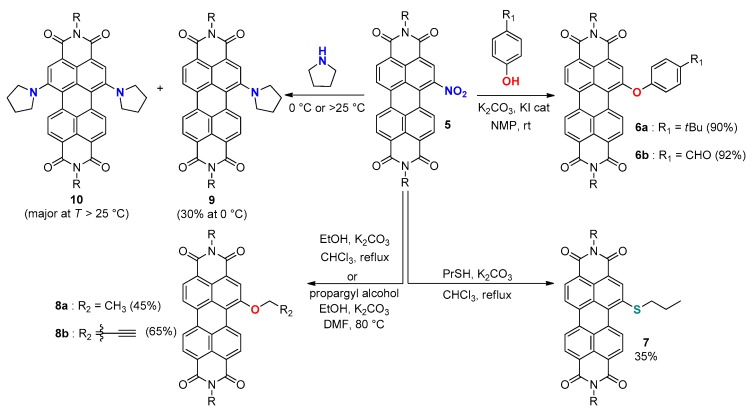
Nucleophilic substitutions from 1-nitroPDI **5**. R = cyclohexyl.

Thanks to the strong electron-withdrawing character of nitro group, nucleophilic substitution reactions can be easily achieved from 1-nitroPDI **5** (Scheme 2). X. Kong et al. described smooth substitutions of 1-nitroPDI with phenol derivatives, ethanol, propanethiol and pyrrolidine as nucleophiles [55]. When stirring at room temperature with 4-tertbutylphenol in the presence of K_2_CO_3_ and a catalytic amount of KI in NMP, the reaction afforded the desired product **6a** in 90% yield. The reaction can occur in DMF or CHCl_3_ in lower yields (less than 65%). Similar conditions were also described to replace the nitro group by electron poor phenoxy and aliphatic ether and thioether in CHCl_3_ or DMF [48,49], which required higher temperature when using aliphatic thiol or alcohol. Remarkably, stirring 1-nitroPDI in neat pyrrolidine at 0 °C already promoted the nucleophilic substitution to give **9** in 30% yield, which highlights the high reactivity of the nitro group toward S_N_Ar [55]. Nevertheless, when the reaction was carried out above 25 °C, the major product surprisingly formed was 1,6-disubstituted PDI **10** without any further discussion from the authors justifying the presence of this side product.

### 3.2. Access to Core-Extended Annulated PDI

The incorporation of annulated-heteroatoms such as S [56], Se [46], or N [50], in the bay positions of the PDI core is recognized to be one of the most effective method to decrease its electron affinity as well as to reduce the intermolecular aggregation between PDI units [57]. In this respect, important research effort has focused on the synthesis of heteroatom-annulated PDIs for the elaboration of high performance organic semiconductors and organic solar cells. To the best of our knowledge, 1-nitroPDI **5** is the most convenient starting material to reach those structures.

**Scheme 3:**
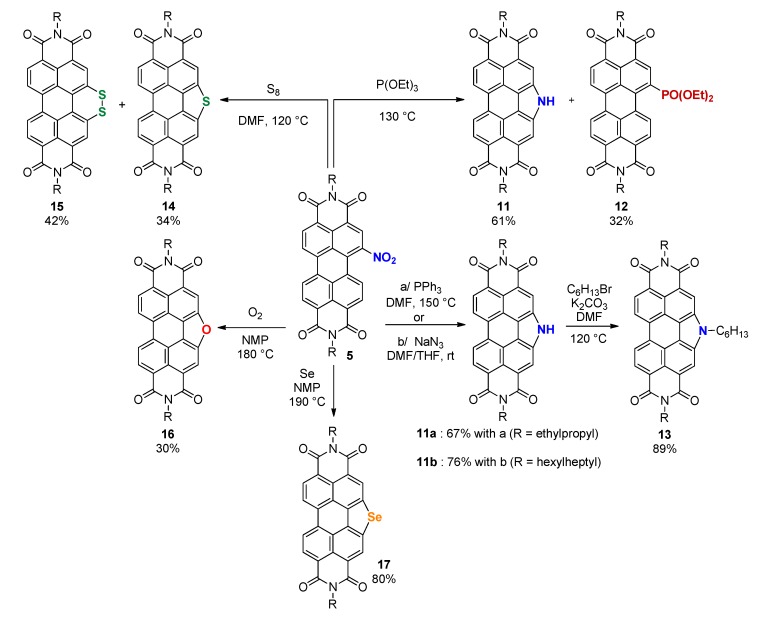
Mono-heteroatom-annulation from 1-nitroPDI **5**. R = cyclohexyl.

Pioneering results were obtained by H. Langhals et al. to reach five-membered rings S- and N-annulated PDIs [38]. The reaction between nitroPDI **5** in neat triethylphosphite, known as Cadogan cylization, yielded to a mixture of carbazole fused PDI **11** and phosphorylated PDI **12** (Scheme 3). The reducing agent promoting the formation of the nitrene intermediate can indeed give rise to a competitive nucleophilic substitution of the nitro group. The protocol was later improved by G. C. Welch and colleagues, who replaced the phosphorous ester by triphenylphosphine in DMF affording desired product **11a** in 67% yield [50]. This yield was considerably increased (91%) when using microwave irradiation in *o*-dichlorobenzene at 180 °C for 2 h [58]. An interesting alternative to this method, which does not require high temperature or generate phosphine oxide, was found by serendipity in our group. Adding a small excess of sodium azide to nitro-PDI in a THF/DMF mixture at room temperature afforded the N-annulated PDI **11b** in 76% yield [33]. We assume that a nucleophilic substitution first occurs followed by the formation of a nitrene intermediate, which spontaneously led to the carbazole ring. This N-containing ring allows the introduction of an extra solubilizing chain using an alkyl halide, or the functionalization with another chromophoric unit to reach a dyad [59]. or the dimerization of N-annulated PDI with a linker via a Buchwald-Hartwig coupling [58].

Moreover, in the original work of H. Langhals, the authors also described the use of sulfur on nitroPDI **5** in DMF or NMP, leading to a mixture of two sulfur containing heterocycles **14** and **15** in different ratio depending on the solvent and the temperature. Notably, at a high temperature in DMF, only thiophene-annulated PDI **14** was formed in 71% yield [38]. L. Chen et al. reported the formation of derivatives **14** and **15** in 34% and 42% yields respectively, by heating the reaction in DMF at 120 °C [60]. On the other hand, bubbling O_2_ or adding selenium powder into 1-nitroPDI **5** in NMP at 180–190 °C led to O-annulated PDI **16** [61]. or selenophene-heterocycle **17** in 30% and 80% yields, respectively [46]. Those syntheses were also applied to the bay-annulation of the perylene tetraester by S. Achalkumar and colleagues [34]. Besides, the authors nicely reported the effect of the heteroatom on the molecular self-assembly.

**Scheme 4:**
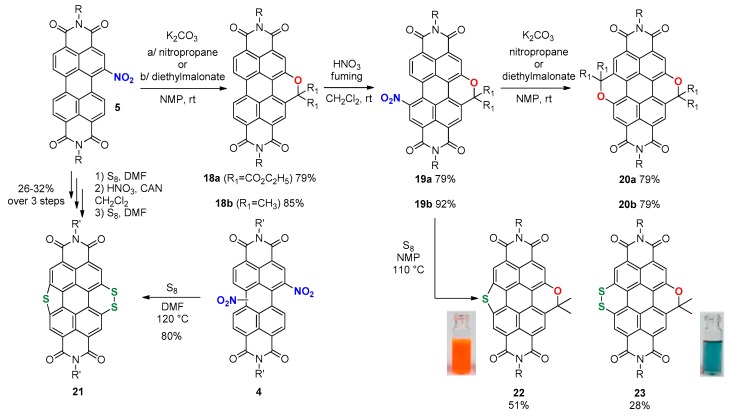
Bis-heteroatom-annulation from nitroPDI derivatives. R = cyclohexyl, R′ = 1-ethylpropyl. Pictures adapted with permission from reference 62. Copyright (2018) John Wiley and Sons.

The possibility of introducing two alkyl chains to enhance the solubility of heteroatom-fused PDIs was explored with the synthesis of pyran-annulated PDI compounds (Scheme 4) [32]. Monopyran-fused PDIs **18** were prepared from 1-nitroPDI via a one-pot nucleophilic substitution/cyclization sequence using 2-nitro-propane or diethyl malonate in NMP at room temperature in 85% and 79%, respectively. The nitration of those derivatives led selectively and almost quantitatively to 7-nitro-pyran-fused PDIs **19,** possibly owing to the electron donating character of the pyran. By subsequent cyclization under the same conditions, bispyran-fused PDIs **20** were obtained.

Following the same sequential synthesis, S-annulated PDIs **14** and **15** were nitrated and bis-bridged, affording unsymmetrical S-PDI-2S **21** in both cases in 76% yield over two steps [60]. In the same work, a one-pot synthesis of the same product was also reported from a mixture of 1,6 and 1,7-dinitro-PDIs **4** in 80% yield. In addition, the S and O-doping strategies were mixed, and in this respect, 7-nitro-pyran-fused PDI **19b** was S-annulated in the presence of sulfur powder yielding to the mixture of two sulfur- and oxygen-containing heterocycle PDIs **22** and **23**. Notably, the two derivatives display very different optical properties, as depicted in the pictures in Scheme 4 [62].

**Scheme 5:**
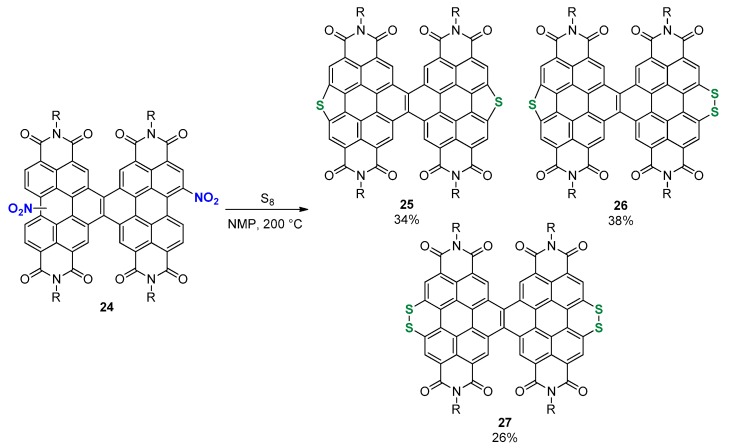
S-annulated fused PDIs. R = 1-pentylhexyl.

Finally, the bis-sulfur-annulation was applied to a fused-PDI dimer **24** through nitration of the parent dimer (Scheme 5) [63]. The reaction with sulfur powder in refluxing NMP afforded a mixture of multisulfur-fused-PDIs **25, 26** and **27**. 

The three products display different optoelectronic properties, and their performances as non-fullerene acceptors (NFAs) in organic bulk heterojunction (BHJ) were demonstrated with power conversion efficiency (PCE) up to 6.9% and high fill factors (>60%).

### 3.3. Palladium-Catalyzed Cross-Coupling Reactions

The bay-decoration of PDI through the formation of C-C bond has been widely employed in organic electronics, such as for the elaboration of OSCs using donor-acceptor (D-A) systems or PDI-based NFAs. The synthetic methodology usually followed is the bromination of PDI in bay position and subsequent Pd-catalyzed cross-coupling reaction. However, as already discussed, the monobromination of PDI suffers from many disadvantages compared to the mononitration (see Section 2).

Recently, the use of nitroarenes as electrophilic partners in Pd-catalyzed couplings for the creation of C-heteroatoms and C-C bonds has been recognized [64]. In particular, the Suzuki–Miyaura coupling (SMC) with nitroarenes has been demonstrated in 2017 by Y. Nakao and S. Sakaki [65,66].

**Scheme 6:**
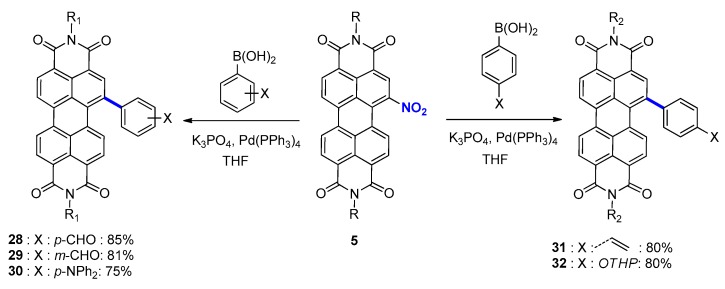
Suzuki–Miyaura coupling reactions from 1-nitroPDI **5**. R = cyclohexyl (R_1_) or 2-ethylhexyl (R_2_).

The first example of a SMC reaction using an electron-deficient arene system such as PDI **5** bearing a nitro group as the electrophilic coupling partner was reported in our group [51]. This reaction uses a straightforward procedure with 4-formyl or 3-formyl phenylboronic acid in the presence of Pd(PPh_3_)_4_ and K_3_PO_4_ as a base in refluxing THF, affording **28** and **29** in 85% and 81% yields, respectively (Scheme 6). Subsequent 1,3-dipolar cycloaddition using C_60_ was carried out on 1-(3-formylphenyl)PDI **29** derivative. The versatility of this original SMC reaction was demonstrated by replacing the phenyl boronic acid substituted with the electron-withdrawing formyl group by the (4-diphenylamino)phenyl electron-donating group. In 2019, J. K. Kallistsis and co-workers applied the same conditions to functionalize the PDI with a protected phenoxy and a styryl moiety [67]. Those anchoring groups were subsequently used to attach withdrawing quinoline derivatives through Heck coupling or nucleophilic substitution after the alcohol deprotection.

**Scheme 7:**
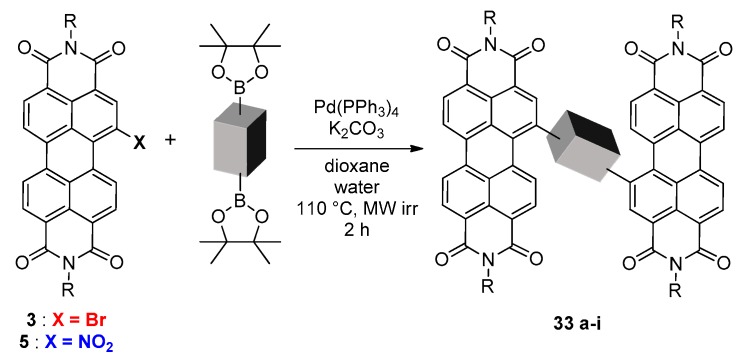
PDI multimerization through SMC from 1-nitroPDI and 1-bromoPDI. R = 1-hexylheptyl.

**Figure 2:**
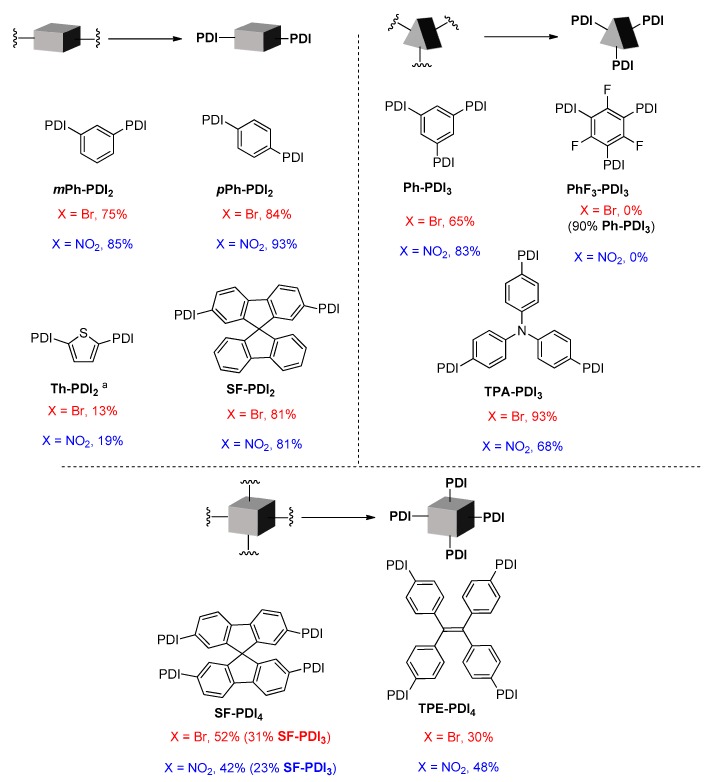
Schematic structures of PDI-based multimers obtained through SMC from 1-nitroPDI **5** and 1-bromoPDI **3**. Figure adapted with permission from reference 57. Copyright (2019) John Wiley and Sons.

Besides, over the last decade, PDI-based multimers have received as much attention as NFAs in organic photovoltaics (OPV) owing to their three-dimensional structure preventing the formation of aggregates and in this respect enhancing the performance of OSCs. In our group, we developed conditions of multimerization through poly-SMC using an aromatic linker with multiple boronic acids or esters and 1-nitroPDI or 1-bromoPDI as electrophilic partners [33]. The described methodology employed Pd(PPh_3_)_4_ as the Pd(0) source with K_2_CO_3_ in a dioxane/water mixture under microwave irradiations, affording various dimers, trimers and tetramers (Scheme 7 and Figure 2). In general, the yields are comparable, starting from nitro **5** and bromo **3** PDI derivatives, and are notably good for the phenylene (Ph) series (over 90% per coupling from 1-nitroPDI).

**Scheme 8:**
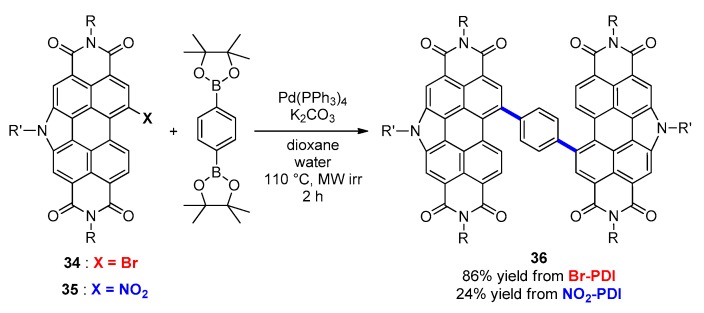
N-annulated PDI multimerization through SMC from 1-nitroPDI and 1-bromoPDI. R = 1-hexylheptyl; R′ = octyl.

However, when the multimerization conditions were applied to enriched *N*-annulated bromo- or nitro-PDIs **34** and **35** in the presence of bis-boronate phenylene, significantly different reaction behaviors were observed (Scheme 8). The reaction carried out with bromo derivative **34** was clean and complete, whereas when applied to nitro-PDI **35**, the product **36** was obtained in 24% yield with 40% of starting material **35** recovered. This result coupled with theoretical calculations suggests that the limiting step of the coupling, the oxidative addition, is favored with the bromo derivative. Nevertheless, the electron-deficiency of the PDI core seems to promote this step, as no significant difference was noticed between the two electrophilic derivatives **3** and **5** during the parent PDI multimerization. In this respect, other Pd-catalyzed cross-couplings using nitro-PDI can be expected to be soon developed for the preparation of unprecedented PDI-based materials. 

## 4. Reduction of the Nitro Group and Functionalization of AminoPDI

Nitroarenes are classically used as key intermediates for the synthesis of aniline derivatives obtained by reduction. Such a transformation of 1-nitroPDI into 1-aminoPDI can be performed using different procedures, affording another interesting building block to design original materials for applications in organic electronics. This transformation and subsequent developments will be presented in this section.

### 4.1. Preparation 1-aminoPDI from 1-nitroPDI

**Scheme 9:**
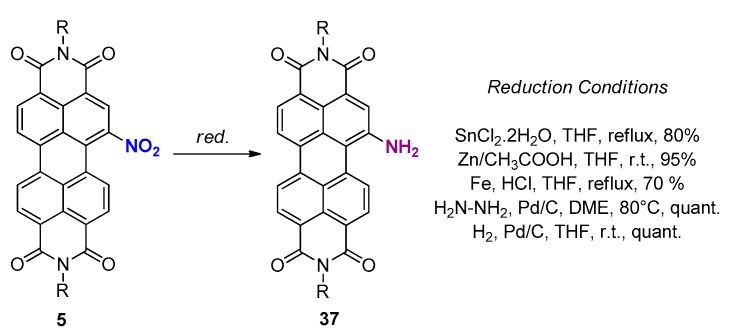
Reduction of 1-nitroPDI **5**.

Langhals and colleagues reported first the reduction of 1-nitroPDI **5** into 1-aminoPDI **37** using iron powder and hydrochloric acid in either ethanol or THF in 81% yield, along with an hydrogenation procedure catalyzed by Pd on carbon with triethylammonium formate as an hydrogen source (Scheme 9) [38]. However, the reactant of choice to perform the reduction of the nitro group into the corresponding amino group in PDI series remains tin (II) chloride dihydrate (SnCl_2_.2H_2_O) in the refluxing of THF, affording the amine with yields of around 80% [30,31,41,42,44,45,47,68]. Alternatively, reduction involving metallic Zn and acetic acid in THF led to 1-aminoPDI in 95% yield [42], whereas metallic iron in the presence of HCl in THF gave the derivative in 70% yield [39]. Another common protocol for reducing nitroarenes to arylamines is the catalytic hydrogenation on Pd/C using hydrazine as an hydrogen source in DME at 80°C, which quantitatively afforded 1-aminoPDI derivative **37** [69]. This method could also be adapted to the direct use of hydrogen gas in THF at room temperature, but Q. Zhang and colleagues noted that *N*,*N′*-(dicylohexyl)1-aminoPDI had to be directly engaged in the following steps without further purification, as instability in air was observed [70]. 

1,6-and 1,7-dinitroPDIs could also be reduced into diaminoPDI using similar procedures. SnCl_2_ was used to transform 1,7-dinitroPDI into diamino derivative in 82% yield [27].

AminoPDI derivatives display a broad charge-transfer band in absorption accompanied by partial or total quenching of their fluorescence and show strong solvatochromism. They are key intermediates for the synthesis of alkyl-aminoPDI derivatives, metal complexes and the preparation of bay-extended PDI architectures, providing sensors and NIR-emitters for examples.

### 4.2. Reactivity of 1-AminoPDI

**Scheme 10:**
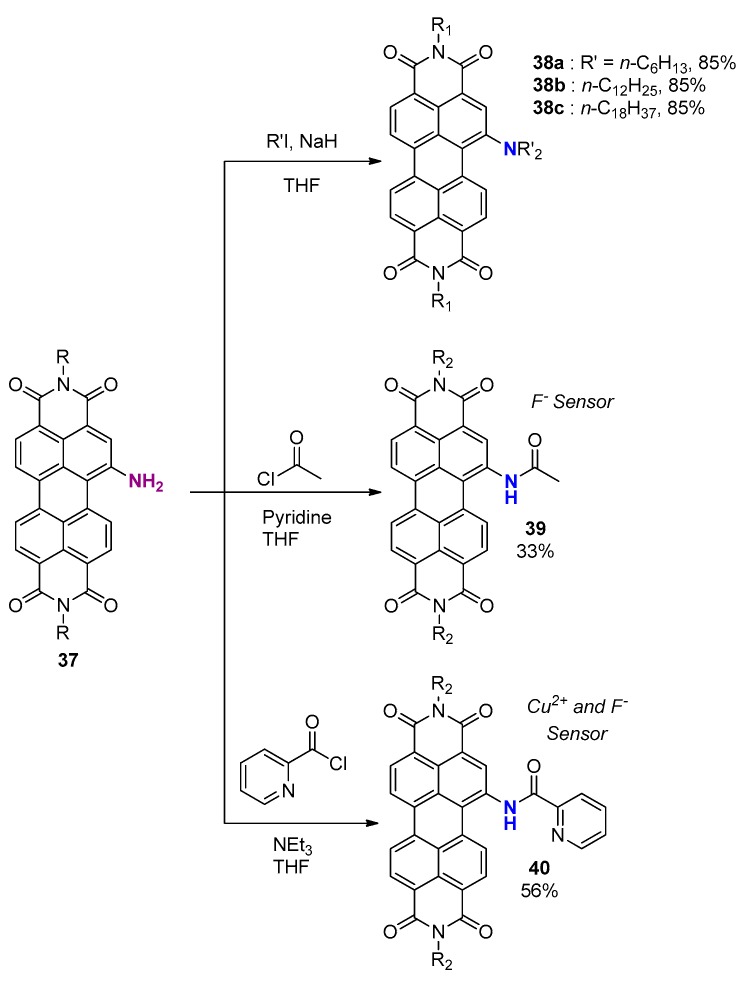
Alkylation and acylation of aminoPDI. R_1_ = 1-ethylhexyl, R_2_ = cyclohexyl.

The alkylation of the amino group was reported using NaH as a base followed by the addition of alkyl iodide providing dialkylated aminoPDI **38a** in an 85% yield (Scheme 10) [30]. Acylation of the amino group was also carried out in only 33% yield using acetyl chloride and pyridine in THF [42], or 2-pyridyl acyl chloride in the presence of Et_3_N to afford fluorescent and colorimetric sensors **39** and **40** for Cu^2+^ and F^-^ ions—in which charge transfers do not occur. In the presence of F^-^ anions, deprotonation of the amide seems to take place, whereas in the presence of Cu^2+^, a dimeric complex is formed. In both cases, this leads to a progressive extinction of the fluorescence and red-shift of the absorption maximum [43].

**Scheme 11:**
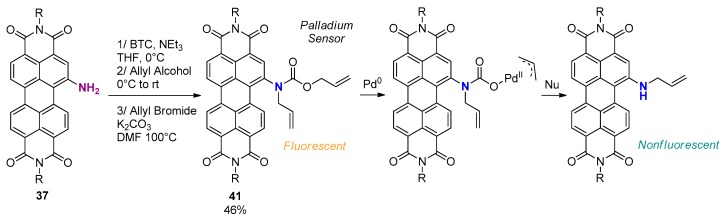
Preparation of a colorimetric and fluorescent Pd^0^ probe. R = 2-ethylhexyl.

Treatment with bis(trichloromethyl) carbonate (BTC) in the presence of triethylamine in THF followed by the reaction with allyl alcohol led to the formation of the fluorescent mono-carboxylated amino intermediate (Scheme 11). The latter was further alkylated with allyl bromide to afford a colorimetric and fluorescent sensor for Pd^2+^ detection in 46% yield [71]. In the presence of Pd^0^, decarboxylation of compound **41** led to the formation of a non-fluorescent species, which also shows its absorption maximum shifting toward longer wavelength.

**Scheme 12:**
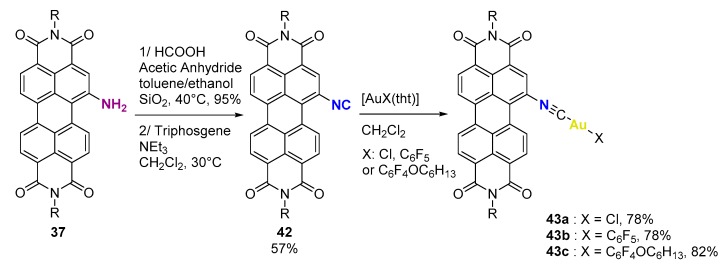
Preparation of 1-isocyanidePDI **42** and its complexation with Au(I). R = 1-ethylpropyl.

Isocyanide derivative **42** also became a target of choice for its ability to form metal complexes, and for being a precursor of carbene complexes. The synthesis of 1-isocyanidePDI required the formylation of 1-aminoPDI **37** with formic acid in 95% yield followed by its dehydration using triphosgene and triethylamine in CH_2_Cl_2_, leading to desired compound **42** in 57% yield (Scheme 12) [39]. Corresponding gold carbene complexes **43** were prepared by coordination of the isocyanide group with Au(I) precursors in very mild conditions. Multi-nuclear architectures were also prepared and some of them were processed as stable Langmuir–Blodgett films.

**Scheme 13:**
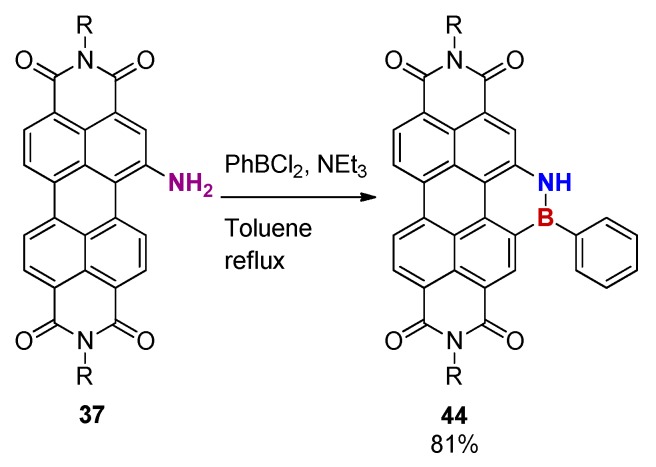
B-N annulation between 1-aminoPDI **37** and dichlorophenylborane. R = cyclohexyl.

Besides, a peculiar azoborine-annulated PDI architecture was reported by Q. Zhang and coll as a highly selective fluoride anion sensor (Scheme 13). This first B-N annulation was performed in 81% yield by reacting 1-aminoPDI with dichlorophenylborane in refluxing toluene and using triethylamine as a base [70]. Compound **44** is an efficient F^-^ sensor, showing extinction of its fluorescence and red-shifted absorption maximum with increasing concentration of anion. This boron fused PDI was also used as an emitter in OLED devices, emitting in the red region.

**Scheme 14:**
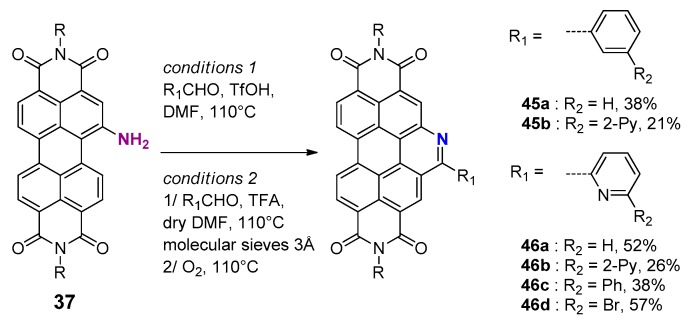
Preparation of azabenzannulated PDI via a Pictet-Spengler approach. R = 2,6-diisopropylphenyl.

A combination of the Pictet–Spengler reaction and subsequent oxidative re-aromatization was reported as a way to access azabenzannulated PDIs **45** and **46** and *N*-decorated coronene bisimides derivatives (Scheme 14). Phenyl (42%) and 2-pyridyl (37%) groups were introduced by Z. Wang and colleagues by reacting aminoPDI **37** with the corresponding aldehyde in the presence of triflic acid (TfOH) in DMF at 110 °C [69]. This transformation was also performed by the group of F. Würthner using trifluoroacetic acid (TFA) as the catalyst [72]. In this reaction, an iminium undergoes an intramolecular electrophilic aromatic substitution and subsequent oxidative re-aromatization under an oxygen atmosphere. These compounds are characterized by low LUMO levels, making them good electron acceptors, and their absorption maximum being blue-shifted compared to regular PDI. Interestingly, PDI-pyridyl based iridium and ruthenium complexes were prepared and characterized; in particular, their phosphorescence in the near-infrared region [73,74].

**Scheme 15:**
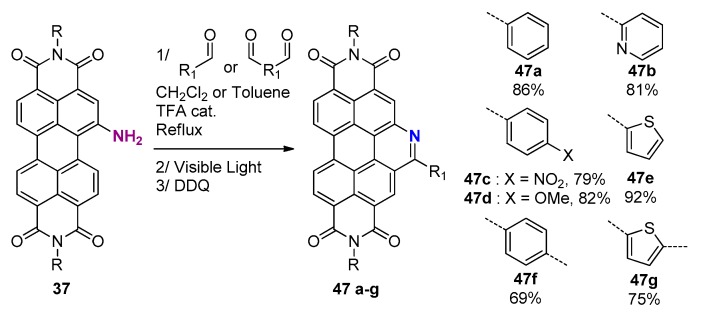
Preparation of azabenzannulated PDI via an imine photocyclization. R = 1-hexylheptyl.

Recently, our group reported an alternative to the Pictet–Spengler reaction to access azabenzannulated PDIs **47** (Scheme 15) [35]. This original reaction involves the formation of an imine intermediate, its photocyclization by visible light followed by re-aromatization using DDQ. These three steps can be performed in one pot process affording azabenzannulated PDI dyes, including dimers, in higher yields than those reported before. 

## 5. Conclusions

In this review, we have highlighted the synthetic use of 1-nitroPDI to modify the bay region of the perylenediimide (PDI) backbone and engineer unlimited photoredox modifications for various applications. Until now, PDI decorated with bromine atom in position 1 occupied a central role in the molecular design of a core-substituted-PDI based light-harvester and electron acceptor because of the versatility of the reactions involving aromatic halides. Nevertheless, as discussed in the manuscript, many reaction types have been now successfully applied to nitroPDI, from nucleophilic substitution, which could lead to an extension through annulation of the perylene core, to palladium catalyzed cross-couplings (Suzuki–Miyaura). This makes the derivative as attractive as bromoPDI. Besides, we have clearly demonstrated here the easier access to 1-nitroPDI (short reaction time, quantitative yield, green and fast purification) due to the higher selectivity of the mononitration compared to monobromination. Considering the two transformation steps, the synthetic methodology involving the nitration of PDI appears more appealing and should be more considered by the community, especially in regard to the possible industrialization.

Moreover, the classical reduction of the nitro group into amino can be easily applied. This group has not been widely exploited so far and the recent efficient imine formation and its subsequent photocyclization provides useful access to new azabenzannulated PDI based materials, promising candidates as non-fullerene acceptors (NFAs) in organic photovoltaics (OPV).
